# Ataxia in Childhood: Epidemiological, Clinical and Neuroradiologic Features, and the Risk of Recurrence

**Published:** 2017

**Authors:** Mohsen JAVADZADEH, Masoud HASSANVAND AMOUZADEH, Shaghayegh SADAT ESMAIL NEJAD, Ezatollah ABASI, Abbas ALIPOUR, Mohsen MOLLAMOHAMMADI

**Affiliations:** 1 Pediatrci Neurology Research Center, Shahid Beheshti University of Medical Sciences, Tehran, Iran; 2 Pediatric Neurology Department, Mofid Children Hospital, Faculty of Medicine, Shahid Beheshti University of Medical Sciences, Tehran, Iran; 3 Neurology and Neuroscience Research Center, Qom University of Medical Sciences, Qom, Iran; 4 Pediatric Department, Mofid Children Hospital, Shahid Beheshti University of Medical Sciences, Tehran, Iran; 5 Pediatric Department, Faculty of Medicine, Urmia University of Medical Sciences, Urmia, Iran; 6 Department of Epidemiology, Faculty of Health, Shahid Beheshti University of Medical Sciences, Tehran, Iran; 7 Hazrat Fatemeh Masoumeh Pediatric Hospital, Qom University of Medical Sciences, Qom, Iran.

**Keywords:** Ataxia, Children, Recurrence

## Abstract

**Objective:**

This study was conducted on the demographic data, clinical characteristics, electroencephalography, neuroradiological findings, and their impact on the recurrence of ataxia.

**Materials & Methods:**

A 3-yr retrospective review of 49 children with ataxia in Mofid Children Hospital, Tehran, Iran was conducted from Apr 2013 to Apr 2016.

The demographic, clinical and paraclinical data were recorded in pre-prepared questionnaires. The patients were also classified in two groups of with or without recurrence and the results were compared. The diagnostic etiologies in our patients were classified as brain tumor, drug ingestion, encephalitis, post infectious immune-mediated disorders, pseudoataxia, trauma, congenital malformations of the central nervous system and hereditary ataxias.

**Results:**

Forty-nine children with ataxia were enrolled. The mean age of the patients with a recurrence of ataxia was more than those without a recurrence.

Neurodevelopmental delay in patients with recurrence was more frequent than those without a recurrence. Abnormal findings in the neuroimaging were seen more in the patients with recurrence than those without recurrence. The most common cause of ataxia in patients with recurrence was hereditary ataxia and in patients without recurrence was a viral post infectious disorder.

**Conclusion:**

After a mean follow-up period of 16.36 months (range: 2-37 months), 9 cases (18.4%) showed recurrence. Older age, abnormal neuroimaging, and neurodevelopmental delay should be considered as the risk factors of recurrence of ataxia in children.

## Introduction

Disturbances in the fine control of posture and movements of the body are called ataxia in which an abnormal gait is the initial and the most prominent feature. When a cerebellar vermis pathology exists, the patient is not able to have a sitting position and head bobbing occurs. On the other hand, the cerebellar hemispheres pathologies cause the patient’s body has the tendency to change direction ipsilateral to the affected hemisphere and also the patient has dysmetria and hypotonia in the same limbs. Bifrontal lobe disease symptoms and signs may be similar to those of cerebellar disease (1).

On the other hand, loss of sensory input to the cerebellum caused by peripheral nerve or posterior column disease, obliged constant looking at the feet to know their location. The gait is wide-based too but is not so much staggering as careful. Moreover, the feet in each step raise a significant height and then slap down by force. In this situation, the gait and station are considerably worsened with the eyes closed and the patient may practically fall called Romberg sign. Sensory ataxia more likely causes difficulty with fine finger movements than with reaching objects (1).

Cerebellar disease also causes a characteristic speech of variable volume and increased separation of syllables (scanning speech), tremor, limb and ocular dysmetria, as well as hypotonia (1). As children are in a developing and learning stage of motor competency, therefore, any kind of ataxia may affect their motor skills, so debilitates them (2). The two most common causes of acute and recurrent ataxia among children are drug ingestion (mostly alcohol, barbiturates and antiepileptic drugs) and acute postinfectious cerebellitis (1).

When a child is referred to physicion, with acute or recurrent ataxia, it is important to determine the exact underlying cause of the ataxia because some of them are really debilitating or fetal and early diagnosis can save patient’s life. These underlying causes are brain tumor, drug ingestion, encephalitis, genetic disorders, migraine, postinfectious disorders, progressive cavitating leukoencephalopathy, pseudoataxia, trauma, vascular disorders (cerebellar hemorrhage and Kawasaki disease) and conversion reaction (1). 

The most prevalent causes of postinfectious cerebellitis are viruses such as coxsackievirus, rubeola, and varicella (3). On the other hand, we should have migraine, encephalitis affecting brain stem and neuroblastoma in our mind as the next steps of our diagnostic studies (1). 

Tumors of the cerebellum (vermal or hemispheric) or tumors adjacent to brainstem particularly pontine gliomas, congenital malformations such as Chiari malformation or Dandy-Walker malformation, and hereditary ataxias usually present as chronic or progressive type of ataxia (1).

The hereditary ataxias which are one of the main causes of chronic ataxia are often diagnosed by longterm symptoms, positive familial history, abnormal gait and muscle weakness, abnormal body tone and abnormal DTR (Deep Tendon Reflex), pes cavus and sensory deficits (4).

Hereditary ataxias are a group of ataxia divided into two groups: autosomal dominant inheritance and autosomal recessive inheritance (Including Abetalipoproteinemia, ataxia telangiectasia, Friedreich`s ataxia, ataxia with episodic dystonia, ataxia without oculomotor apraxia, Hartnup disease, juvenile GM2 gangliosidosis, juvenile sulfatide lipidoses, Maple syrup urine disease, Marinesco-Sjogren syndrome, Pyruvate dehydrogenase deficiency, Ramsay Hunt syndrome, Refsum disease and respiratory chain disorders) (1).

Pseudoataxia called epileptic ataxia is a kind of seizure which its presentation is ataxia and other gait disturbances. The patient may appear inattentive or confused. The lack of nystagmus proposes that ataxia is a seizure manifestation and not caused by drug toxicity (1).

The worldwide prevalence of childhood ataxia (both genetic and acquired causes) is estimated to be 26 per 100000 children (5).

Previous studies in Iran have mostly focused on adult population and have not reviewed all of the most common causes of ataxia. Since ataxia is a major cause of admissions in the Neurology Ward of Mofid Children Hospital, and because an extensive study on children was not done in Iran, this study was carried out to evaluate the demographic, clinical, and paraclinical characteristics of ataxia in childhood, and their effect on the rate of recurrence.

## Materials & Methods

From Apr 2013 to Apr 2016, 49 children under 18 yr old with the initial complaint of ataxia were admitted to the Neurology Ward of Mofid Children Hospital, Tehran, Iran. The demographic data, the history of presenting illness, the results of the physical examination and electroencephalography (EEG) and neuroimaging findings were recorded in pre-prepared questionnaires. 

The recorded data was studied retrospectively and the patients were classified into different groups according to their final diagnosis. Telephone interviewing was done carefully in order to complete the patients’ recorded information and we asked about the recurrence of their disease.

Among the patients admitted with ataxia and were followed up after discharge by telephone interviewing and outpatient visits, those who had recurrent symptoms led to re-admission due to its intensity and severity considered as recurrence of ataxia. The data collected in the questionnaire was also classified in two groups of with or without recurrence and the results were compared between two groups. 

The data was recorded confidentially and informed consent was obtained from parents. Each case was characterized by a numerical code.

The demographic, clinical, EEG and neuroimaging data of each child were reviewed in detail, such as age, sex, history of neurodevelopmental delay, the clinical course (acute, recurrent, chronic static, chronic progressive), EEG report, findings of neuroimaging, final diagnosis, the recurrence of the disease, and the time interval between recovery and recurrence. Magnetic resonance imaging (MRI) and EEG study were performed for all patients reported by an expert neuroradiologist and pediatric neurologist, respectively.

The final diagnoses as the cause of ataxia were classified as brain tumor, drug ingestion, encephalitis, postinfectious immune-mediated disorder, pseudoataxia, trauma, congenital malformation and hereditary ataxia.

The quantitative variables are indicated by mean (and standard deviation) and qualitative ones by numbers (and percentage). Mann-Whitney U-test was used to compare between quantitative variables in different groups and Chi-Square test for qualitative variables. 

Whenever necessary, the Fisher Exact test was used.

P values less than 0.05 were considered as statistically significant and the data analysis was done by SPSS Ver. 22.0 (Chicago, IL, USA) and Stata (V12) Statistical software.

## Results

Forty-nine children with ataxia were enrolled in terms of demographic, clinical and paraclinical data (Table 1). 

The patients with pseudoataxia were diagnosed using EEG and, their seizure episodes were certainly

confirmed. We had seven cases with hereditary ataxia. Four cases were diagnosed as ataxia telangiectasia and three as Friedreich ataxia.

Nine cases (18.4%) had recurrence of ataxia (Table 1). The mean interval between the first recovery and the recurrence of symptoms was 7.55 months. Ataxia recurrence incidence rate per 1000 person-months was 12.55 (95%CI: 6.53-24.12).

The mean age of the patients with recurrence of ataxia (90 months) was more than patients without recurrence (45.5 months). The calculated difference was also meaningful according to statistical studies (P=0.003).

Multivariate analysis using Cox’s regression model, after controlling the effects of sex, clinical course, neurodevelopment and EEG findings, showed that the risk of ataxia recurrence was increased 1.03 times by increasing every month of patients’ age (95%CI=1.009- 1.041). Female to male ratio in patients with recurrence was more than patients without, but the difference was not statistically meaningful (P=0.15). No relation was found between the clinical course of the ataxia and its recurrence (P=0.74).

Six cases (66.7%) among those with recurrence and 7 (17.5%) among those without recurrence had abnormal findings in neuroimaging which was statistically meaningful (P=0.003). After controlling the effect of sex, clinical course, neurodevelopment and EEG findings, multivariate analysis using Cox’s regression model demonstrated that abnormal neuroimaging increases the risk of ataxia recurrence 4.76 times (95% CI= 1.1-20.58).

Neurodevelopmental delay in patients with recurrence (55.6%) was more than those without recurrence (20%) and the mentioned difference was significant (P=0.03). 

Abnormal EEG was reported in 44.4% of the patients with recurrence and 25% of the group without recurrence which was not statistically meaningful (P=0.24).

The most common cause of ataxia in patients with recurrence was hereditary ataxia (44.4%) and in

patients without recurrence was post infectious disorders (57.5%).

## Discussion

This research is one of the few studies reviewed the epidemiological, clinical and paraclinical data of children with ataxia in two groups with recurrence and without recurrence in parallel.

The mean age of the patients in our study was 53.67 months, similar to other studies (6, 7). However, the mean age of the children was significantly higher in patients with recurrent ataxia (90 months) than those without recurrence (45.5 months). This difference seems to be due to more etiologies that are benign at younger ages, for example, post infectious immunemediated disorders.

Among all 49 patients, 65% were male matched with other studies (7-9).

In our study, the most common presentation form of ataxia was the acute ataxia seen in 67.3% of the cases, again in compatibility with another study (10). Thirteen (26.5%) patients had abnormal results of neuroimaging. Previous studies have reported mixed results (from 0 to 74 percent) in this respect (6, 9, 11, 12). In our study, the abnormal neuroradiological findings were much more frequent in patients with a recurrence than those without it (66.7% vs. 17.5%).

**Table 1:** Comparison of the features of patients according to the Recurrence

**Variables**		**Recurrence**		**Total** (N=49) n (%)	***P*** **- Value**
		**No (N =40)** **n (%)**	**Yes (N =9)** **n (%)**		
**Age (month)** **Mean(standard deviation)**		45.5(31.3)	90(44.6)	53.67(37.7)	0.003
**Sex** **(female/male)**		12/28	5/4	17/32	0.15
**Clinical course**	**Acute**	31(77.5)	2(22.2)	33(67.3)	0.74
	**Recurrent**	2(5)	5(55.6)	7(14.3)	
	**Chronic static**	5 (12.5)	0(0)	5(10.2)	
	**Chronic** **Progressive**	2(5)	2(22.2)	4(8.2)	
**Abnormal neuroimaging**		7(17.5)	6(66.7)	13(26.5)	0.003
**Abnormal Development**		8(20)	5(55.6)	13(26.5)	0.03
**Abnormal EEG**		10(25)	4(44.4)	14(28.6)	0.24
**Diagnosis**	**Brain tumor**	2(5)	0(0)	2(4.1)	NA*
	**Drug ingestion**	3(7.5)	0(0)	3(6.1)	
	**Encephalitis**	2(5)	1(11.1)	3(6.1)	
	**Genetic disorders**	1(2.5)	1(11.1)	2(4.1)	
	**Post infectious**	23(57.5)	2(22.2)	25(51)	
	**Pseudo ataxia**	2(5)	0(0)	2(4.1)	
	**Trauma**	1(2.5)	0(0)	1(2)	
	**Congenital** **Malformations**	3(7.5)	1(11.1)	4(8.2)	
	**Hereditary** **Ataxias**	3(7.5)	4(44.4)	7(14.3)	

* NA: not applicable

Therefore, all patients who experience recurrent attacks of ataxia, regardless of the severity of symptoms and the time interval between the attacks, should undergo meticulous brain imaging (preferably MRI) to find any clue that could help the physician to discover the etiology of ataxia.

In our study, 26.5% of the patients had the history of neurodevelopmental delay, similar to another study (13). This delay was more common in patients afflicted by recurrent ataxia than those who were not (55.6% and 20%, respectively). Such a finding may reflect the deleterious effects of underlying cause of ataxia on acquisition of cognitive and motor skills in growing children. Thus, it is critical to begin rehabilitation programs in these patients to overcome potential disabilities, as soon as possible.

Fourteen (28.6%) of our cases showed abnormality in their EEG. Another study, reported it as 67%, which the difference may be caused by the various sample size of each study (14).

Postinfectious ataxia (51%) and hereditary ataxia (14.3%) were the most common causes of ataxia diagnosed in our study. Previous studies also showed similar results (6, 10, 11). The former was most common in patients without recurrence, and the latter in patients with recurrent ataxia.

In our research, among nine patients with recurrence, one of them was diagnosed as enteroviral encephalitis and had recurrence after 2 yr. In some studies, ataxia was one of the main clinical manifestations of enteroviral infection (15).

A case of episodic ataxia type 2 was reported that we mentioned it as a kind of genetic disorder. Two cases had recurrence after viral infection and one case of recurrence was diagnosed finally as Dandy-walker malformation mentioned as congenital malformation in Table 1. Four cases with hereditary ataxia had recurrence, two of them were ataxia telangiectasia and 2 were Friedreich ataxia.

All patients at discharge had a complete recovery and symptoms of ataxia were greatly relieved to the extent that they were able to walk without help. This was reported already as improvements, mostly in acute ataxia (16).

**In conclusion, **after a mean follow-up period of 16.36 months (range: 2-37 months), 9 cases (18.4%) had recurrence and no mortality was reported. In this study, the epidemiologic, clinical and paraclinical characteristics of the patients were compared in two groups of with or without recurrence. A comparison between the two groups based on having or not having recurrence in our study showed the age, abnormal neuroimaging and neurodevelopmental delay as the main risk factors of recurrence for ataxia in children. 

Finally, we suggest more studies that are comprehensive in order to expand our knowledge of the ataxia in children.
